# Why we should care about asylum seeking Latina pregnant migrants

**DOI:** 10.3389/frcha.2025.1665928

**Published:** 2025-11-06

**Authors:** Maria Cuervo, Amanda Venta

**Affiliations:** Department of Psychology, University of Houston, Houston, TX, United States

**Keywords:** Latina, immigrant, pregnant women, trauma, Latinx, hispanic

## Abstract

Latina immigrants seeking asylum in the U.S. report high levels of trauma exposure and posttraumatic distress. Our clinical and research experiences highlight the prevalence of these health disparities for pregnant migrants, with serious consequences for them as well as for the developmental trajectory of their children. This minireview focuses on asylum seeking Latina pregnant migrants, and the implications current polices, and circumstances have on their health and wellbeing as well as their children's health and well-being.

## Introduction

Although exact numbers are unknown, estimates suggest that thousands of women were pregnant or became pregnant while they wait to seek asylum at the U.S.-Mexico border (1). Pregnant migrants are highly vulnerable to trauma (2), with serious implications for the course of the pregnancy and maternal health, including risks of complications and heightened psychological distress (3). These risks extend beyond pregnancy, shaping children's later development through both biological pathways (e.g., prenatal stress exposure) and postnatal caregiving environments (4). This commentary focuses on asylum seeking Latina pregnant migrants, and the implications current polices, and circumstances have on their health and wellbeing as well as their children's health and well-being.

Our research team has collected data from more than 600 Latinx immigrants seeking asylum at the U.S./Mexico border—bringing together expertise from clinical psychology and public health. Although this piece is not meant to reflect all of their experiences, and we recognize significant heterogeneity among Latinx immigrants, we bring this significant expertise and context to this manuscript. We are immigrants and Latinxs ourselves and have heard hundreds of personal narratives from asylum seekers, publishing quantitative, qualitative, and narrative accounts of their struggles and survivorship. But most existing literature does not include the stories of the many women who are pregnant or have given birth during migration.

Isabel (pseudonym to protect the individual's privacy) was one such woman. She was a young, twenty-year-old woman from Honduras who traveled to the U.S. pregnant and with her five-year-old son, two-year-old daughter, and husband. Her family left Honduras due to gang persecution, extreme violence, and poverty. Many times on their journey, she did not think they would make it. While on a bus with her family in Mexico, men with guns came aboard and demanded payment. Her two-year-old daughter became sick and dehydrated during their migration, and the family was forced to stay back, separated from the rest of their traveling companions, waiting for their daughter to recuperate. On several occasions, her husband would forgo what little food they had to ensure that she and their children had enough to eat. The journey was horrific; they waited in Mexico for months just to receive an appointment with U.S. officials to seek asylum, and she felt terrified for her family and herself at each step of the way. During our interview, she broke into tears recounting the horrors and exclaiming, “pero llegamos muy tarde” [“we arrived too late”]. Isabel felt unwell and was experiencing extreme pain the day she and her family appeared before U.S. officials to make their claim for asylum. She felt disoriented, like she was in a dream, but knowing, even in that state, that something was wrong. The family was admitted to the U.S., to await a years-long immigration court process, and Isabel was taken to a hospital in south Texas. Her baby had died. She was given medication to induce labor. This tragedy occurred just one day before she found herself sitting in a large, fluorescent-lit cafeteria being interviewed by our research team. She felt grief and guilt; she questioned whether her baby would have survived if they had stayed in Honduras, moved more quickly during migration, had access to better travel conditions, or gotten an asylum appointment earlier. We questioned to what extent immigration policies were to blame for her heartbreak and the loss of this precious life.

Isabel's story, among others we have heard, shed light on a tragic reality—asylum-seeking women are carrying children, have lost children, and are waiting far too long to exercise their right to seek asylum. These women are in danger. Prior literature has reported on the migration journey itself and how it exposes women to multiple dangers that elevate their risk for perinatal mental health disorders (2). Women who are forcibly displaced often endure conflict, disasters, and other mass violence events, including witnessing the deaths of family members, sexual assault, and rape (2). These traumatic exposures, combined with intimate partner violence—which can intensify during pregnancy and is more common in conflict-affected settings—create cumulative layers of risk for depression, PTSD, and anxiety (2). Additionally, these premigration experiences are compounded during transit by dangerous migration routes, homelessness, and poverty, which generate ongoing instability and uncertainty (3). The process of displacement is not only destabilizing but also marked by profound uncertainty, loss, and fear, all of which compound psychological vulnerability during the perinatal period (2, 3). Such exposures help explain the disproportionately high prevalence of depression and PTSD among forced migrants compared with economic migrants, with perinatal PTSD affecting nearly one in five women in forced migration contexts (3). Migration-specific risk factors such as insecure residency status, trauma exposure, and fear of deportation further compound these dangers, underscoring the direct link between migration trajectories and mental health outcomes (3).

In addition to trauma exposure, migrant women frequently face structural barriers that limit access to medical and mental health care during pregnancy and postpartum (2). Indeed, as emphasized by prior authors research and policy often treat migrants as a homogeneous group, neglecting the unique vulnerabilities of forced migrants, particularly asylum seekers with insecure residency (2). This lack of recognition translates into insufficiently tailored services and systemic inequities in perinatal health support. As a result, many women at the highest risk of depression, PTSD, or anxiety are least likely to receive adequate care, reinforcing cycles of unmet need and poor outcomes for both mothers and children (2). Moreover, many do not have access to medical care throughout their journey and instead experience unimaginable traumas and hardship. Pregnancy is a sensitive period for elevated health risk, and a mother's exposure to adversity and trauma increases this vulnerability for both the mother and child (5, 6). Migrant mothers also face additional stressors which contribute to mental and physical health risks, such as lack of social support, marital strain or a lack of marital support, difficulties adjusting to the host country, socioeconomic difficulty, stress and mental health issues, and uncertainty given their legal status (3, 7). Even after being granted provisional entrance to the U.S., many migrant pregnant women may be unwilling or unable to seek help or may not be aware of available services due to cultural, linguistic, and economic barriers as well as fear that pending immigration court proceedings or documentation status may be negatively affected (7, 8). Prior literature underscores that a lack of social support is one of the most consistent and consequential risk factors for perinatal mental health difficulties among migrant women (2, 3). Indeed, Stevenson and colleagues (3) indicate that poor social support strongly predicts depression, anxiety, and PTSD, with the loss of social networks during migration leaving women isolated and vulnerable, particularly when compounded by insecure immigration status and fear of deportation (3). Similarly, Rees and Fisher (2) highlight that inadequate support—whether from partners, families, or communities—is closely linked to poor mental health outcomes, often intersecting with intimate partner violence and functional impairment that hinder adjustment and settlement (2). Together, these findings emphasize that pregnant migrants, who already face disproportionate trauma and instability, are further disadvantaged by inadequate formal and informal support, underscoring the urgent need for culturally sensitive interventions, peer networks, and systemic resources that can mitigate isolation and promote perinatal wellbeing (2, 3).

Health care professionals should engage in advocacy for vulnerable immigrant clients (9). Pregnant migrant women often face isolation, trauma, and barriers to care, but everyday people can play an important role in supporting them. Simple acts of inclusion—such as inviting women into community groups, neighborhood gatherings, or parenting circles—can help reduce loneliness and foster belonging. Offering practical assistance with transportation to prenatal appointments, childcare for older children, or navigating local systems can ease daily burdens that feel overwhelming in a new country. Bridging language and cultural gaps is also crucial, whether by helping interpret paperwork, practicing conversational language, or explaining how healthcare and social services work. Just as important is providing emotional support: listening without judgment, validating their experiences, and creating a safe space for sharing. Community members can also connect women to available resources, such as local clinics, food programs, or mental health hotlines, which they may not know how to access. Finally, advocating for inclusivity in schools, healthcare, and community settings, and challenging stigma or discrimination when it arises, helps create safer environments. Taken together, these everyday actions can strengthen protective social networks and buffer against the risks of depression, anxiety, and trauma that migrant women face during pregnancy and beyond.

Researchers should partner with this community to understand their needs and shine a light on these needs in order to address lacking healthcare services and support the mental health of migrant mothers during the vulnerable pre- and perinatal periods. Additionally, researchers can play a vital role in supporting pregnant migrant women by addressing the systemic gaps highlighted above. Firstly, as Stevenson and colleagues (3) previously argued it is important to design studies that disaggregate data between forced and economic migrants, ensuring that the unique vulnerabilities of refugees and asylum seekers are not obscured by broad categories. This includes focusing on understudied outcomes such as PTSD, grief, and separation anxiety, alongside depression and anxiety (3). Second, scientists can prioritize longitudinal and community-based research that tracks women's mental health across the perinatal period and beyond, moving beyond cross-sectional snapshots to capture trajectories of risk and resilience (3). Third, researchers can ensure that their work is culturally and linguistically adapted, including validating screening tools in diverse languages, collaborating with community organizations, and incorporating women's voices in study design. Fourth, by conducting implementation and intervention studies, researchers can identify what forms of social, peer, and systemic support are most effective in improving outcomes. Finally, scientists can act as advocates, translating findings into actionable recommendations for policymakers, healthcare providers, and community organizations, and pushing for policies that guarantee equitable access to perinatal and mental health services. In doing so, researchers not only generate knowledge but also help dismantle systemic barriers and promote health equity for migrant women and their children.

Lastly, healthcare professionals are uniquely positioned to support pregnant migrant women because they are often the first point of contact during the perinatal period (10). Providing effective care starts with routine, trauma-informed, and culturally sensitive screening for depression, anxiety, PTSD, and related conditions, while ensuring that language barriers are addressed through interpreters or translated materials. Professionals can also offer continuity of care by coordinating across maternity, mental health, and social services, recognizing that fragmented systems often leave migrant women without sustained support. Building trust and rapport is essential—listening respectfully, validating women's experiences, and avoiding stigmatizing language can help reduce fear, especially for asylum seekers who may worry about deportation or discrimination. Healthcare providers can also serve as advocates, linking women to community resources such as peer support groups, food programs, or housing assistance, and helping them navigate complex health systems. Additionally, training in cultural humility equips professionals to better understand the social and historical contexts of forced migration, enabling them to recognize and respond to the intersecting effects of trauma, poverty, and isolation. By combining clinical care with advocacy, healthcare professionals can play a critical role in reducing systemic barriers, strengthening protective supports, and improving perinatal outcomes for migrant women and their families. Furthermore, healthcare professionals need to be aware of stressors and hardships faced by migrant mothers and conduct careful screening and follow-up. Routine gynecological screening in this group should include pertinent information on immigration variables—like duration of residence in the host country, adversities survived during the pregnancy, language fluency, living conditions, social and marital support—and avoid probing legal status, as it deters women with uncertain immigration status from seeking care. And as Americans, we must advocate for speedy processing for migrant mothers and families at our Southern border and increased medical and mental health access regardless of political affiliations. We should collectively call for policies that address the stressors pregnant migrant women face, such as discrimination, poverty and social isolation, especially as we commence a new election cycle with campaigns that are increasingly hostile towards migrants.

Although our work helps highlight Isabel's story a limitation of our study is that the work that brought us to conduct research at the border was not particularly investigating maternal mental health, pregnancy, or the perinatal period. Rather, while conducting work at the border we realized the gravity of the issue. Our hope is that this research piece inspires further research, policy, and clinical work with this population.

## Data Availability

The original contributions presented in the study are included in the article/Supplementary Material, further inquiries can be directed to the corresponding author.
